# Direct spectroscopic speciation of the complexation of U(VI) in acetate solution

**DOI:** 10.1007/s00706-014-1278-6

**Published:** 2014-07-24

**Authors:** Günther Meinrath, Dorota Kwiatek, Zbigniew Hnatejko, Stefan Lis

**Affiliations:** 1 RER Consultants, Fuchsbauerweg 50, 94036 Passau, Germany; 2 Department of Rare Earth, Faculty of Chemistry, Adam Mickiewicz University, Grunwaldzka 6, 60-780 Poznan, Poland

**Keywords:** UV–Vis spectroscopy, Absorption spectra, Coordination chemistry, Stability constant

## Abstract

**Abstract:**

As a result of systematic UV–Vis absorption spectroscopy studies in the U(VI) acetate system, the single component spectrum of [UO_2_CH_3_COO]^+^ with characteristic parameters was evaluated and applied in quantitative deconvolution of multicomponent spectra. Free acetate concentrations were obtained by the use of geochemical and probabilistic modelling codes. A total of 51 UV–Vis spectra were collected in a wide range of experimental conditions where coordination of U(VI) by acetate ion was indicated by characteristic variations in the spectra structure as compared to UO_2_^2+^. Using chemometric data analysis, the resulting factor structure was evaluated to obtain a subset of 14 spectra holding only one coordinated species next to UO_2_^2+^_(aq)_. The molar absorption coefficient for the U(VI) monoaceto species was estimated as *ε*_418_ = 17.8 ± 1 dm^3^ mol^−1^ cm^−1^. Spectral deconvolution was used to obtain an estimate of the species concentrations which allowed to calculate for each sample the free acetate concentration, the total U(VI) amount and, eventually, to estimate the formation quotient lg *β*_11_ = 2.8 ± 0.3 of UO_2_(CH_3_COO)^+^.

**Graphical Abstract:**

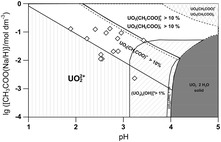

## Introduction

Acetate, CH_3_COO^−^, is the salt of the monoprotic acetic acid, CH_3_COOH. The protonation constant *K*_A_ of acetic acid is reported to be p*K*_A_ = 4.76 [1, 2] in diluted aqueous solutions at 298 K. Under those conditions, hexavalent uranium occurs exclusively as linear dioxo entity, UO_2_^2+^ [3]. This dioxo cation is almost always coordinated in the plane equatorial to the axial uranyl oxygens by four, five, or six neighbours. Coordination of the uranyl(VI) ion by acetato ligands may occur in a rather limited range between pH 1.9 and pH 5.0. At values below pH 1.9, the free acetate concentration is too low for significant U(VI) coordination, even in acetate brines. At values above pH 5, the solubility of U(VI) with respect to UO_3_·2H_2_O is limiting the U(VI) concentration except at total acetate concentrations >10^−2^ mol dm^−3^. Here, however, complexation by atmospheric CO_2_ may interfere because of the very high coordination tendency of U(VI) and CO_3_^2−^ [4]. Experimental speciation of U(VI) should consider the onset of hydrolysis at about pH 3 [5], which may interfere. To circumvent, both precipitation and hydrolysis of U(VI) high concentrations of acetate are required in solution.

Considering the narrow field of stability of U(VI) acetate species, a surprising amount of work has been devoted to the study of the U(VI) acetate interaction, both on the experimental [6–12] and the theoretical level [13–17]. The literature given is but a selection from the past decade and the abundance available. Three groups can be distinguished: crystallographic studies [6–8], analytical studies often involving very advanced equipment like synchrotron sources [9–12], and quantum chemical numerical calculations [13–17]. Considering the wide range of experimental techniques applied to the study of U(VI) acetate interaction in aqueous solutions, e.g. infrared and Raman spectroscopy [18, 19], potentiometry and calorimetry [20], capillary electrophoresis [12], X-ray absorption [16, 21], mass spectrometry [13, 15], ion exchange chromatography [22], a standard method of U(VI) speciation in aqueous solution is almost missing: UV–Vis absorption spectroscopy. Some early work is available [23–25], however, without detailed information on single component characteristics like band position and molar absorptions. Görrler-Walrand and de Jagere [26] report a single component spectrum of UO_2_CH_3_COO^+^ without further information on the determination and characteristics of this spectrum. Recently, single component UV–Vis absorption spectra of U(VI) acetato species have been proposed from a factor analysis study, again without further information even on very basic characteristics of the spectra, e.g. molar absorptions. The wavelength range was limited to a rather narrow region of 400–470 nm, thus omitting the characteristic absorptions of U(VI) in the range 340–400 nm [27]. The onset of the strong absorption towards the UV is a characteristic feature for each U(VI) species and a crucial proof for the reliability of proposed single component spectra in peak deconvolution studies. Successful applications of single component U(VI) spectra in the U(VI)-acetate system are not to our knowledge.

Therefore, we have studied the U(VI) acetate system systematically by UV–Vis absorption spectroscopy. For the first time, we report the single component spectrum of UO_2_CH_3_COO^+^ together with its characteristic parameters and show its applicability to the quantitative deconvolution of mixed spectra from the U(VI) acetate system. This data is of basic importance to compare U(VI) acetate complexation with other U(VI) carboxylate interactions up to naturally occurring organic materials where carboxylate groups are considered as functional groups relevant for U(VI) binding [14].

## Results and discussion

**Fig. 1:**
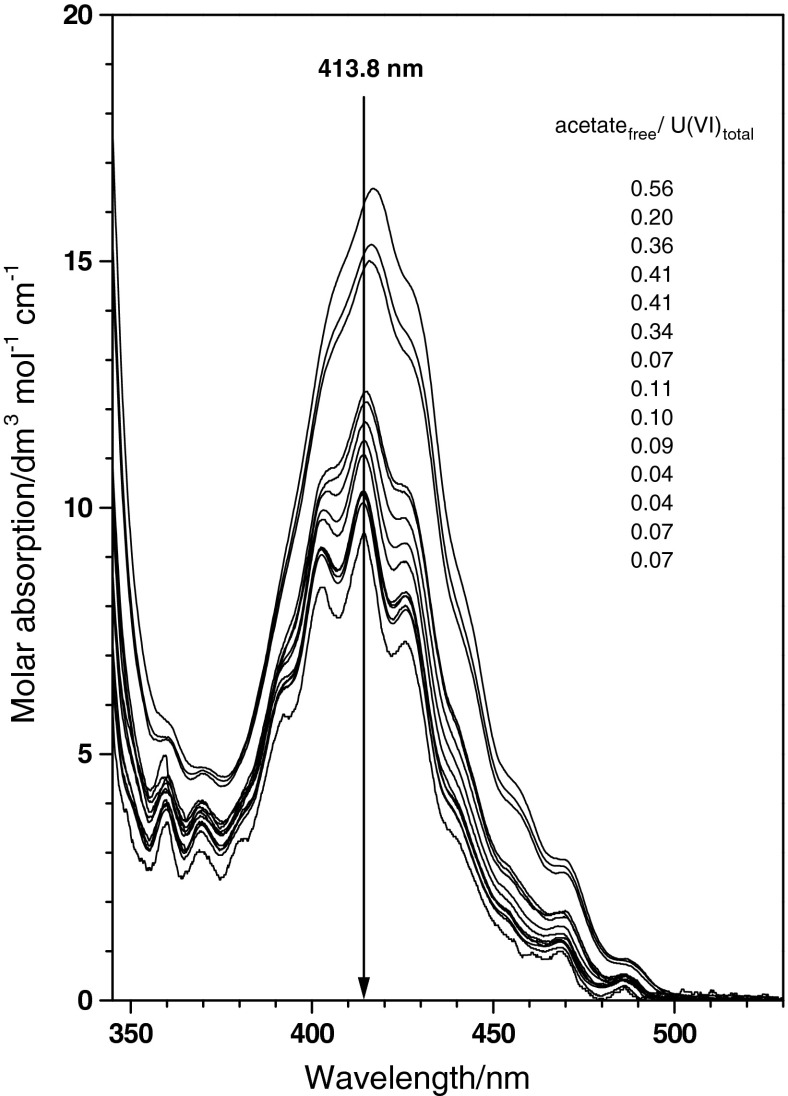
Absorption curves of 14 experimental spectra of U(VI) collected in U(VI) acetate medium at varying pH and total U(VI) and acetate concentrations. Data is normalised by the U(VI) concentration. Sequence of spectra corresponds to the sequence of concentration ratios

A set of 14 U(VI) absorption spectra are given in Fig. 1, normalised to the total U(VI) concentration. This presentation reveals a systematic increase in the molar absorption from 9.7 dm^3^ mol^−1^ cm^−1^ at 413.8 nm to about 16.5 dm^3^ mol^−1^ cm^−1^ at 416.9 nm. A molar absorption of 9.7 dm^3^ mol^−1^ cm^−1^ at 413.8 nm is known for the free UO_2_^2+^ ion in aqueous solutions. The increase in molar absorption in the characteristic band of U(VI) correlates with the ratio of free acetate and total U(VI) concentration, that is, with increasing coordination of UO_2_^2+^ by acetate. The concentration ratio given in Fig. 1 does not vary systematically with the change in the absorption (e.g. at the wavelength of 413.8 nm). Such a systematic change should not be expected. First, the ratio holds the total U(VI) concentration, not the free U(VI) concentration. Second, both the calculated free acetate concentration and the total U(VI) concentration are experimental quantities which are associated with a measurement uncertainty. Forming the ratio of uncertain data further enhances the uncertainty. Nevertheless, the observed absorption and the concentration ratio are strongly correlated (Pearson correlation *r* = 0.8).

No isosbestic point is observed. Coordination of U(VI) by acetate causes a weak band shift to longer wavelengths, barely visible at the most intense spectra in Fig. 1. A hyperchromic effect is intensifying the absorption. Increasing coordination by acetate also causes a bathochromic shift of the steep absorption band in the region below 350 nm.

The UV–Vis spectroscopic study of the U(VI) acetate system is limited by four constraints. The first constraint is the low molar absorption of UO_2_^2+^(aq) in the characteristic absorption bands about 413.8 nm. The second constraint is the onset of U(VI) hydrolysis at values between pH 3.3 and pH 3.7 (depending on the total U(VI) concentration). Third, the acetate ligand is formed from acetic acid with a p*K*_A_ = 4.76 [1, 2].

**Fig. 2:**
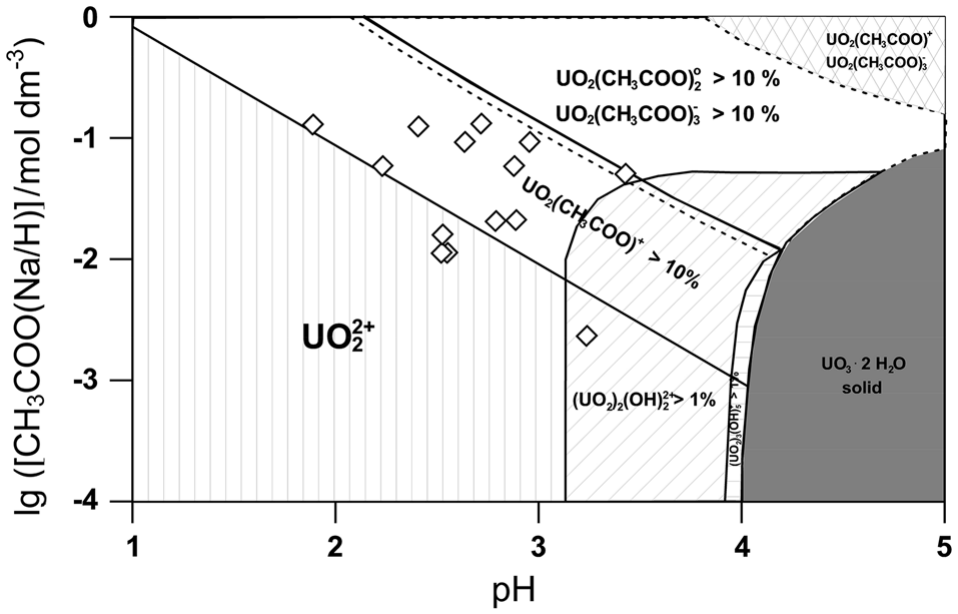
Constraints in the system U(VI) acetate in the range pH 1 to pH 5 and total acetate concentrations varying from 10^−4^ to 1 mol dm^−3^. The diagram is calculated for a fixed total U(VI) concentration of 0.01 mol dm^−3^ from data collected in Table 1. *Diamond-shaped symbols* indicate locations of experimental samples. The varying U(VI) concentrations of these samples does not play a role except in case of oligomeric hydrolysis product formation

Fourth, the solubility of U(VI) in solution is limited by the solid UO_3_·2H_2_O. Figure 2 illustrates these constraints, where stability regions of various species are given on basis of geochemical modelling using data from Table 1. The diamond-shaped symbols indicate the 14 samples where coordination of the free uranyl ion, presumably by acetate, was observed.

**Table 1:** Thermodynamic parameters used for stability field simulation in Fig. 2

Species	Formation constant/acidity constant	References
CH_3_COO^−^	−4.76	[45]
(UO_2_)_2_(OH) _2_^2+^	−6.14	[28]
(UO_2_)_3_(OH) _5_^+^	−17.30	[29, 30]
UO_2_CH_3_COO^+^	2.80	[45]
UO_2_(CH_3_COO)_2_	4.70	[45]
UO_2_(CH_3_COO) _3_^−^	5.10	[45]
UO_2_CO_3_°	9.10	[32]

Figure 2, which is deduced from literature data, suggests that the monoacetato species of U(VI) forms only at quite acidic pH and rather high total acetate concentrations without interference from other species. The stability fields for the acetato species have been selected to represent conditions where the species concentrations are calculated to be above 10 % of the total species concentrations. For the hydrolysis species, the stability fields enclose conditions where species concentrations are above 1 %.

The distinction is due to the difference in molar absorptions. While the oligomeric hydrolysis products (UO_2_)_2_(OH) _2_^2+^and (UO_2_)_3_(OH) _5_^+^have molar absorptions of about 100 dm^3^ mol^−1^ cm^−1^ (421.8 nm) [28] and about 475 dm^3^ mol^−1^ cm^−1^ (429.0 nm) [29], respectively, the acetato species have much lower molar absorptions in the range of the characteristic absorption band of U(VI). Hence, even minor amounts of oligomeric U(VI) hydrolysis products in a sample will affect an experimental UV–Vis spectrum. The respective stability field of a U(VI) diacetato species is represented in Fig. 2 by the region enclosed in dashed lines. In this region of the lg[acetate]-pH diagram, all acetato species considered, UO_2_CH_3_COO^+^, UO_2_(CH_3_COO)_2_°, and UO_2_(CH_3_COO) _3_^−^, are calculated to occur in solution with relative amounts above 10 %. Hence, direct spectroscopic speciation in such complex media requires accurate knowledge of the respective single component spectra of U(VI) acetato species to have a chance to be meaningful. In the cross-hatched region in the upper right corner of Fig. 2, the monoacetato and the triacetato species of U(VI) are prevalent. At values above pH 5, all acetic acid is dissociated and further shift of pH does not significantly increase the amount of acetate ligand at a given total acetate concentration. Furthermore, under atmospheric conditions, carbonate will form in solution from CO_2_ dissolved in the solutions to become a potent competitor for U(VI) [30–32]. Thus, Fig. 2 summarises the essential features of the interaction of acetate with U(VI), against which the experimental findings from this study will be probed.

While concisely illustrating the U(VI)-acetate system, Fig. 2 is not considered as a guide for this investigation. Data evaluation is based, as far as possible, on model-free numerical and statistical models. No decision in this study is made with respect to Fig. 2.

**Fig. 3:**
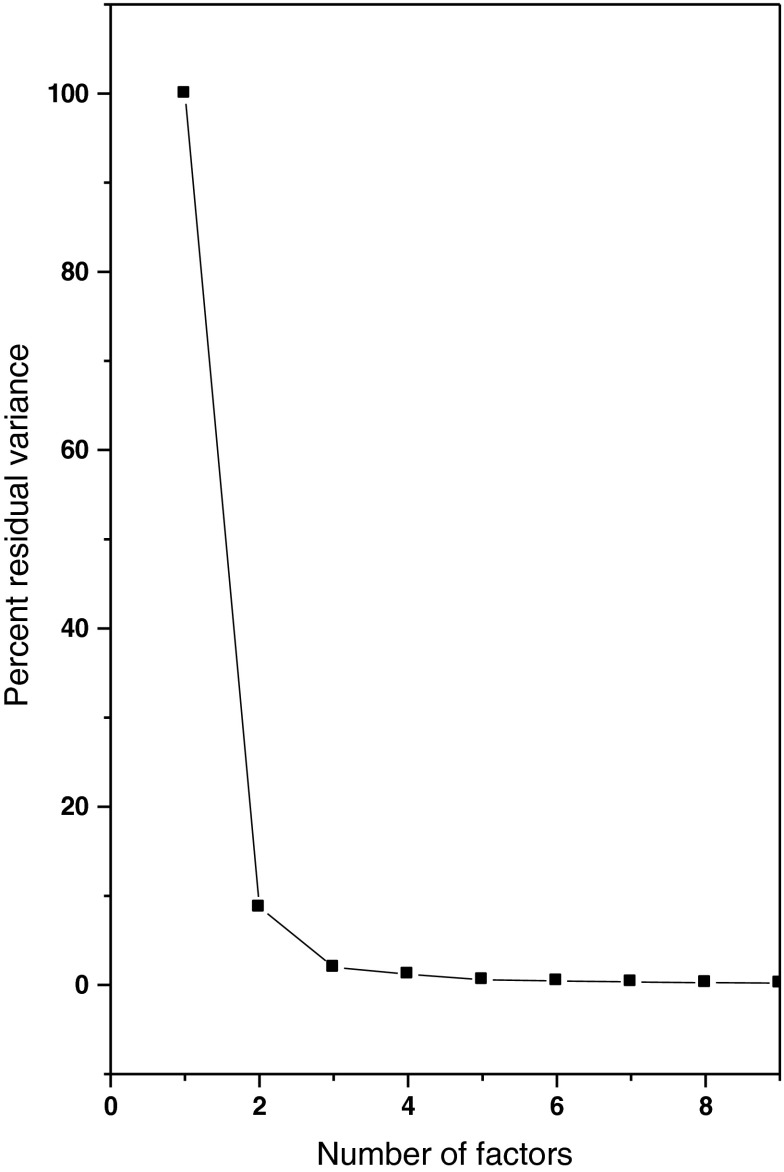
Graphical representation of the SCREE test. The first factor explains about 85 % of the experimental variance, the second about 7 %. The subsequent factors are not able to remove significant parts of the remaining variance

From a total of 51 spectra collected at random in a wider range of conditions, 51 indicated coordinated U(VI) due to significant difference of the observed UV–Vis absorption spectrum from the well-known spectrum of UO_2_^2+^. From these 51 spectra, a set of spectra had to be found holding only UO_2_^2+^ and one further component. A larger number of spectra could readily be eliminated for their physico-chemical state suggesting either influence of hydrolysis or formation of higher U(VI) acetato species. The remaining spectra were selected by either submatrix analysis [33] or computer-assisted target factor analysis (CAT) [34]. Thus, a data set with 14 spectra was obtained with the resulting factor structure given as a SCREE test [35] in Fig. 3. The first factor alone explains more than 90 % of the observed variance. The second factor explains just 7 % of the variance—that is the additional signal caused by the hyperchromic shift due to coordination by acetate.

The remaining factors explain random noise in the spectra contributing in sum less than 2 % of the spectroscopic signal. It has been noted previously that a larger number of statistical criteria have been forwarded to identify the number of significant factors from a principal component analysis [36]. Due to the unavoidable presence of random noise in experimental data, none of these criteria can be unambiguous. The researcher’s choice must be judged on basis of the outcome of the overall analysis. In case of UV–Vis spectroscopic investigations, factor analysis forwards information which may serve as criteria. An example is the total U(VI) concentration in a sample. This concentration is known to the experimenter (within experimental uncertainty). Spectral deconvolution and factor analysis provide an estimate for this concentration by the sum of all U(VI) species quantitatively estimated in solution for all solutions based on the experimental spectra and the single component spectra included into the data analysis. Thermodynamic parameters, e.g. formation quotients of the species formed in solution, may serve as additional criterion.

**Fig. 4:**
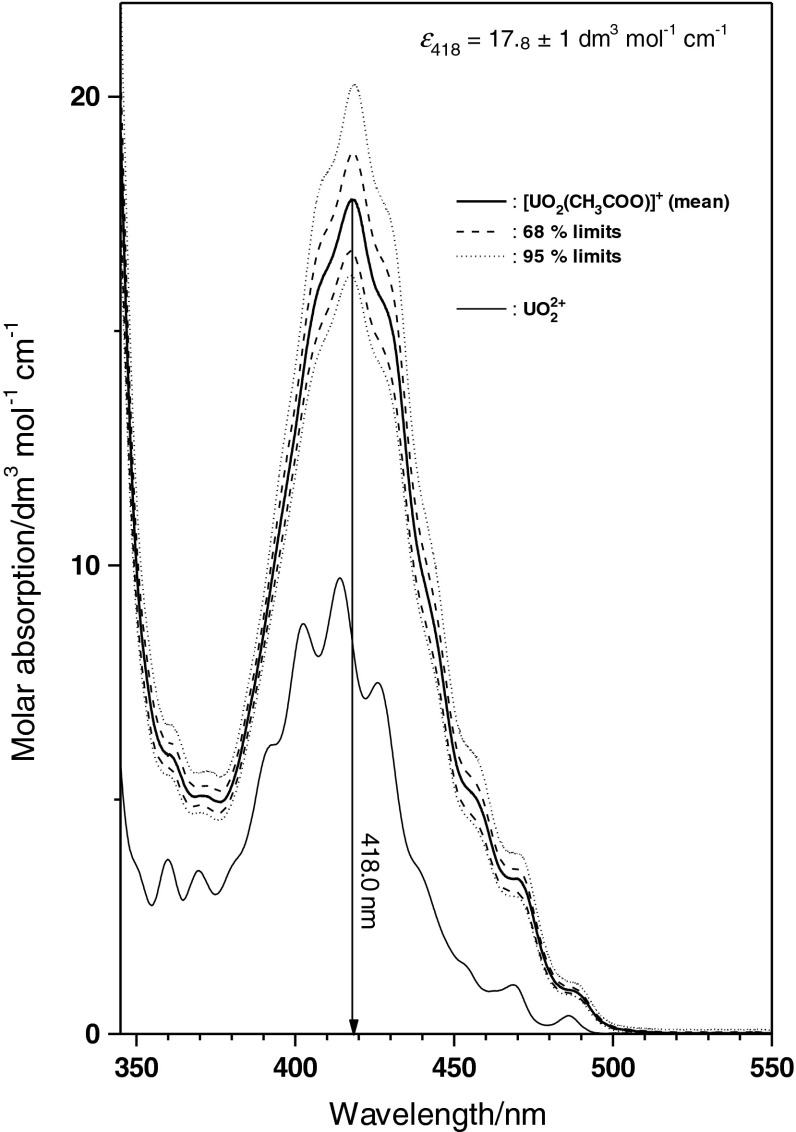
Single component spectrum of UO_2_CH_3_COO^+^, compared to the spectrum of UO_2_^2+^(aq). Absorption maximum of the characteristic band of U(VI) is found at 418.0 nm. Molar absorption *ε*_418_ = 17.8 ± 1.0 dm^3^ mol^−1^ cm^−1^. *Dashed lines* give upper and lower 0.68, *dotted lines* 0.95 ‰ uncertainty obtained from moving block bootstrap analysis

The single component spectrum of UO_2_CH_3_COO^+^ resulting from these analyses is shown in Fig. 4, together with the spectrum of UO_2_^2+^(aq) for comparison. The monoacetato species shows an absorption maximum at 418.0 nm with a molar absorption coefficient *ε*_418_ = 17.8 ± 1 dm^3^ mol^−1^ cm^−1^. The spectrum is given in Fig. 4 together with 0.68 ‰ (dashed) and 0.95 ‰ (dotted), obtained from a moving block bootstrap analysis [36]. Within the limits of precision, the UV–Vis absorption spectrum of UO_2_CH_3_COO^+^ does not show a shift in the absorption maximum or the bands/shoulders in the characteristic absorption region of U(VI) compared to the absorption spectrum of UO_2_^2+^ except in the absorption maximum. The small difference in the position of the absorption maximum of the characteristic band of U(VI) causes the shift observed with the experimental spectra in Fig. 2.

**Fig. 5:**
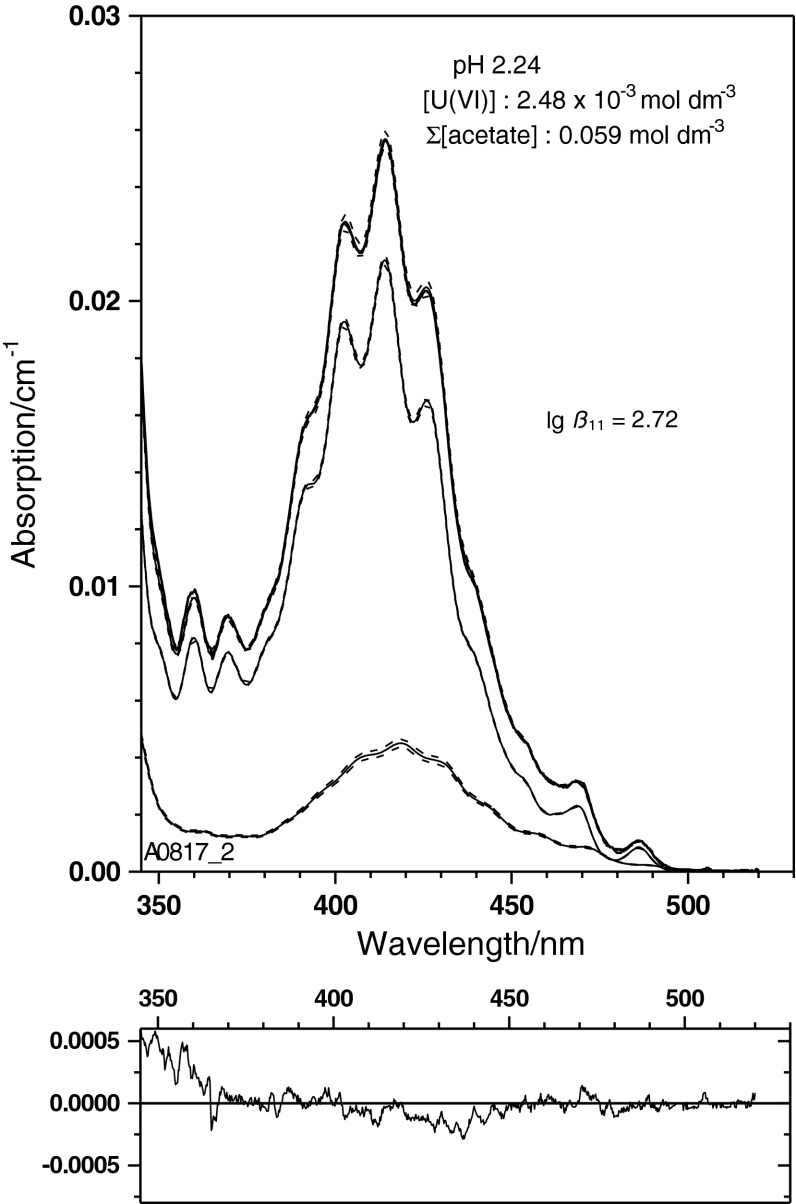
Experimental U(VI) spectrum obtained at pH 2.24 and total acetate concentration (CH_3_COO(H/Na)) of 0.059 mol dm^−3^. Under those conditions, the monoacetato species is a minor component only

**Fig. 6:**
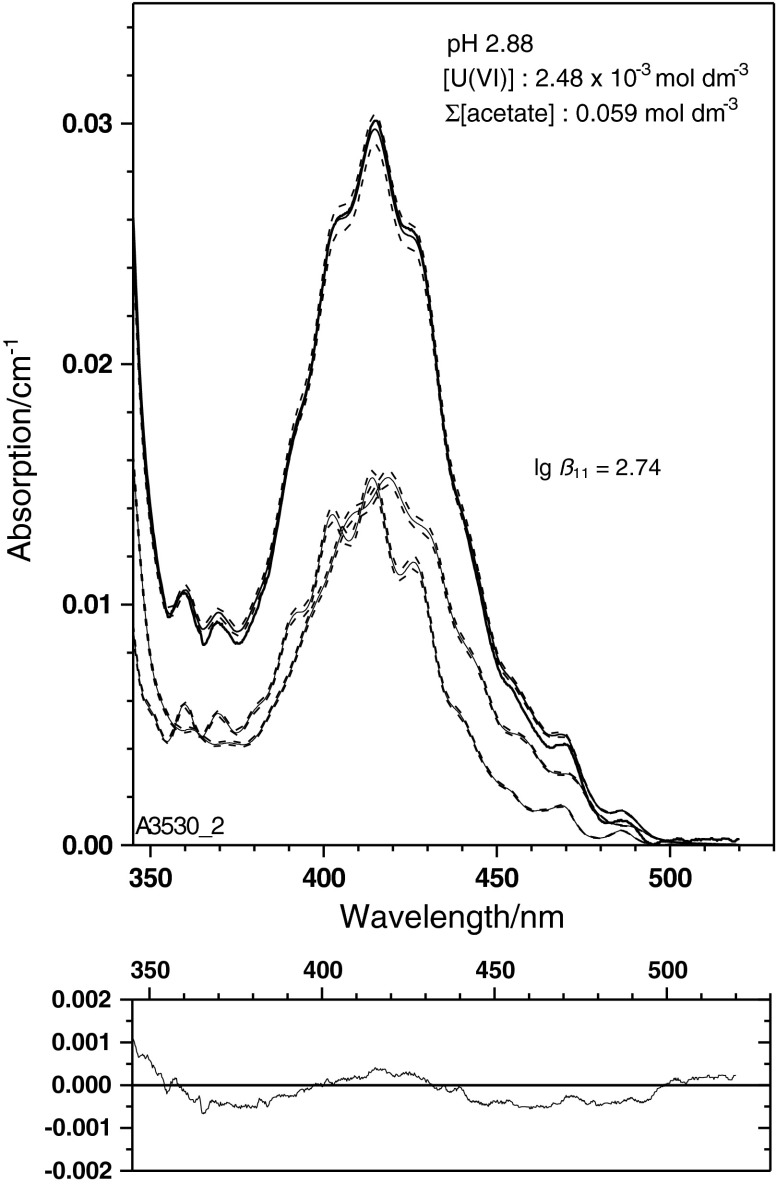
Experimental U(VI) spectrum obtained at pH 2.88 and a total acetate concentration (CH_3_COO(H/Na)) of 0.059 mol dm^−3^. Conditions are shown where both species have an almost equal spectral contribution

**Fig. 7:**
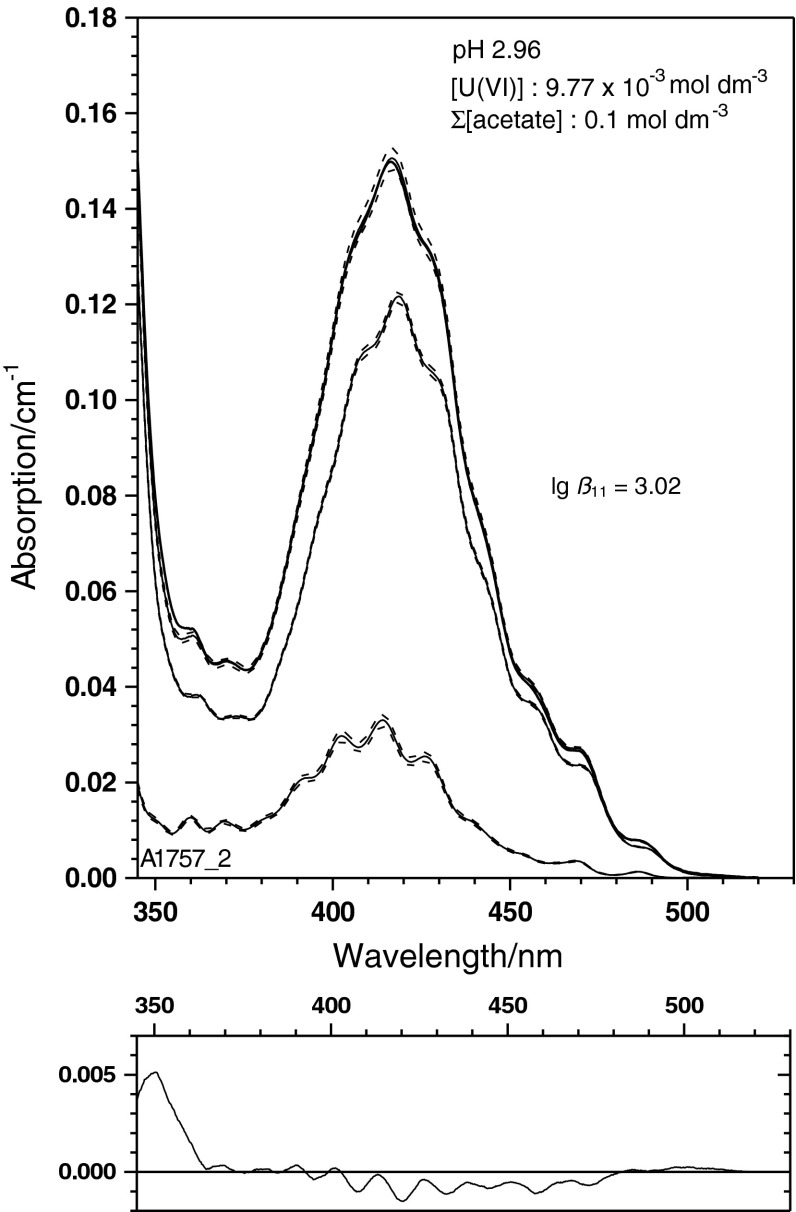
Experimental U(VI) spectrum obtained at pH 2.96 and a total acetate concentration (CH_3_COO(H/Na)) of 0.1 mol dm^−3^. Here, the U(VI) monoacetato species is the predominant spectral component

The single component spectrum of UO_2_(CH_3_COO)^+^ (Fig. 4) is applied to the experimental spectra given in Fig. 1. The physical and chemical parameters of these samples are summarised in Table 2 together with results of a spectral deconvolution using the mean value spectra of UO_2_^2+^ and UO_2_CH_3_COO^+^ from Fig. 4. Examples of this deconvolution are given in Figs. 5, 6 and 7 for three spectra with widely varying ratio of the U(VI) species concentrations. These spectra illustrate the power of the single component spectrum given in Fig. 4 to quantitatively interpret U(VI) solutions with widely varying physico-chemical conditions.

**Table 2:** Results of spectral deconvolutions of 14 UV–Vis using single component spectra of UO_2_^2+^ and UO_2_CH_3_COO^+^

pH	lg [CH_3_COO^−^]_free_	[U(VI)]/mol dm^−3^	[U(VI)]_calc_/mol dm^−3^	∆/%	[UO_2_^2+^]/mol dm^−3^	[UO_2_CH_3_COO^+^]/mol dm^−3^	lg *R*
1.9	−3.629	2.42 × 10^−3^	2.40 × 10^−3^	−0.8	2.16 × 10^−3^	2.39 × 10^−4^	−0.96
2.24	−3.656	2.48 × 10^−3^	2.45 × 10^−3^	−1.2	2.19 × 10^−3^	2.58 × 10^−4^	−0.93
2.42	−3.090	2.42 × 10^−3^	2.42 × 10^−3^	0	1.73 × 10^−3^	6.90 × 10^−4^	−0.397
2.52	−3.979	2.43 × 10^−3^	2.44 × 10^−3^	0.4	2.29 × 10^−3^	1.46 × 10^−4^	−1.20
2.54	−4.127	1.013 × 10^−3^	1.01 × 10^−2^	−0.3	9.49 × 10^−3^	6.00 × 10^−4^	−1.20
2.56	−4.119	1.04 × 10^−3^	9.61 × 10^−4^	−8	9.29 × 10^−4^	3.25 × 10^−5^	−1.46
2.65	−3.006	2.44 × 10^−3^	2.45 × 10^−3^	0.4	1.59 × 10^−3^	8.66 × 10^−3^	−0.26
2.73	−2.759	4.76 × 10^−3^	4.97 × 10^−3^	4.2	1.85 × 10^−3^	3.13 × 10^−3^	+0.23
2.8	−3.582	2.43 × 10^−3^	2.50 × 10^−3^	2.8	2.13 × 10^−3^	3.75 × 10^−4^	−0.75
2.88	−2.996	2.48 × 10^−3^	2.42 × 10^−3^	−2.4	1.55 × 10^−3^	8.68 × 10^−4^	−2.53
2.89	−3.492	4.94 × 10^−3^	5.03 × 10^−3^	1.7	4.01 × 10^−3^	1.01 × 10^−3^	−0.60
2.96	−2.709	9.77 × 10^−3^	1.02 × 10^−2^	4.2	3.35 × 10^−3^	6.86 × 10^−3^	+0.31
3.24	−3.952	2.61 × 10^−3^	2.61 × 10^−3^	0	2.28 × 10^−3^	3.30 × 10^−4^	−0.84
3.43	−2.562	4.88 × 10^−3^	5.02 × 10^−3^	2.8	9.10 × 10^−4^	4.11 × 10^−3^	+0.66

[A], molar concentrations of species A

∆, percentual difference between theoretical and calculated U(VI) total concentration

*R*, concentration ratio of UO_2_CH_3_COO^+^ and UO_2_^2+^

These spectra demonstrate further that the bathochromic shift of the absorption edge to the UV region can be well interpreted quantitatively. These three spectra are the first examples to our knowledge where a single component spectrum for the U(VI) monoacetato species is applied to the deconvolution of experimentally obtained U(VI) spectra in acetate medium.

Table 2 also presents mean value results for the species concentrations from the deconvolution, the calculated free acetate concentrations, the total U(VI) concentrations, the respective U(VI) concentrations as obtained by summing the species concentrations, and a comparison of both U(VI) concentrations in per cent differences.

**Fig. 8:**
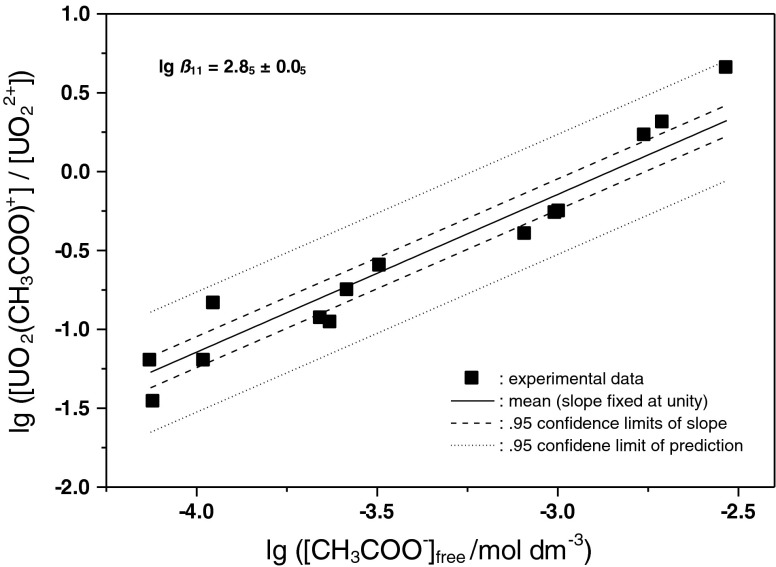
Interpretation of the numerically determined species ratios R by the calculated free acetate (CH_3_COO^−^) concentration. The double-logarithmic plot yields formation constant lg *β*_11_ as the *y*-intercept with the theoretical slope of 1. *Dashed lines* give 0.95 ‰ uncertainty of the slope. The *dotted lines* give 0.95 ‰ uncertainties of predicting a further point

The quotient of species concentrations is obtained from the quantitative spectral deconvolutions, while the free acetate concentrations may be estimated on basis of the known total acetate concentration, the pH, and the p*K*_A_ of acetic acid. These quantities are given in Table 2. A graphical summary is given in Fig. 8 where parameters from Table 2 are interpreted by a trend with fixed slope 1 according to Eq. (1). The formation quotient lg *β*_11_ for UO_2_CH_3_COO^+^ is defined as

1 $$\lg \, \beta_{11} \, = \lg \frac{{[{\text{UO}}_{2} {\text{CH}}_{3} {\text{COO}}^{ + } ]}}{{[{\text{UO}}_{2}^{2 + } ]}} - \lg \, [{\text{CH}}_{3} {\text{COO}}]^{ - }$$

where [A] denotes molar concentrations of species A.

The intercept lg *β*_11_ is obtained as 2.8_5_ ± 0.0_5_, where the uncertainty is given on the 95 % level. The evaluation of a formation quotient has not been a main focus of this study because lg *β*_11_ has been previously determined by a variety of methods rather consistently. Respective presentations are available in literature (e.g. recently [12]). We note that the evaluation of this parameter on basis of the derived single component spectrum Fig. 4 falls within this range of literature data.

In the ionic strength range used here (0.01 < *I* < 0.2), values in the range 2.4 < lg *β*_11_ < 3.0 have been reported. Notwithstanding a complete uncertainty analysis of the thermodynamic data, the reliability of lg *β*_11_ from this study is tentatively estimated as lg *β*_11_ = 2.8 ± 0.3. While the dashed lines in Fig. 8 give the uncertainty of the slope, the dotted lines give the estimated uncertainty (0.95 ‰) of predicting a further measurement value on basis of the existing ones. All experimental data points are found within that limit.

## Conclusions

The 51 UV–Vis spectra of U(VI) acetate in the concentration range from 9 × 10^−4^ mol dm^−3^ to 1 × 10^−2^ mol dm^−3^ and the pH range from 1.9 to pH 5 were registered. From these, a subset of 14 spectra holding only a single complexed species was obtained using submatrix analysis or computer-assisted target factor analysis (CAT) [33, 34]. This procedure is routinely applied to resolve UV–Vis absorption data of complex systems, e.g. [37–39].

The single component spectrum is assigned to the UO_2_CH_3_COO^+^ species. It is the only species to consider under given experimental conditions. In the given range of pH, hydrolysis is the only possible interfering reaction. Great care has been taken to avoid hydrolysis species with amounts above 1 %.

The derived spectrum of the UO_2_CH_3_COO^+^ species has a molar absorption of 17.8 ± 1.0 dm^3^ mol^−1^ cm^−1^ at the absorption maximum of the characteristic absorption band of U(VI) at 418 nm. This spectrum is able to interpret the 14 spectra from which it was derived with only minor residuals. The total U(VI) concentrations in each of the 14 solutions is reproduced within a few percent.

From the relative species concentrations of UO_2_CH_3_COO^+^ and UO_2_^2+^, the formation quotient could be derived as 2.85 ± 0.05, whereby the figure given after the ‘±’ symbol is a 0.95 ‰ range but only accounting for the misfit of the regression line. It is not a complete measurement uncertainty budget.

The deconvolution of the spectra into the species’ individual amounts has been done by a least square residuals criterion. The residuals contribute about 2–3 % of the maximum absorption. With the exception of the spectrum at pH 2.96 given in Fig. 7, the residuals are unspecific. This single spectrum’s residuals show a certain fine structure potentially indicative of a small contribution of another species—even if at the edge of detectability. Clearly, the availability of the UO_2_CH_3_COO^+^ single component spectrum opens the chance to interpret more complex spectra, e.g. those recorded in a range of physical conditions where interference by hydrolysis is possible. Characterisation of hydrolysis components is relevant to avoid misinterpretation as higher acetate complexation. This will be the focus of our future activities.

## Experimental

Total U(VI) concentrations were in the range 9 × 10^−4^ mol dm^−3^ to 1 × 10^−2^ mol dm^−3^. The range of pH was varied between 1.9 and 5.0. Total acetate concentration was varying between 0.003 and 0.14 mol dm^−3^. From these information, free acetate concentrations varying between 7.7 × 10^−5^ and 5.8 × 10^−2^ mol dm^−3^ are obtained from numerical speciation.

U(VI) perchlorate solutions were prepared from UO_2_(CH_3_COO)_2_·2 H_2_O solid (CHEMAPOL/LACHEMA Co., Warsaw, Poland) by dissolution in water and re-precipitation with H_2_O_2_ (20 %). The yellow precipitate was filtered, washed, and heated in a furnace at 200 °C (4 h) and 400 °C (8 h). The resulting UO_3_ solid was redissolved in a stoichiometric amount of perchloric acid (70 %, Fluka Co., Switzerland). A more detailed description of the procedure is given in [40]. The acetate medium was prepared from a standard solution of 0.3 M NH_4_CH_3_COO (POCH S.A. Co., Gliwice, Poland).

Numerical modelling of the sample solutions indicates that ionic strength of the samples varies between *I* = 0.01 and *I* = 0.3, hence is found outside the range of diluted solutions where the thermodynamic parameters become highly sensitive to small changes of composition. All experimentation, if not stated otherwise, was made at room temperature.

### Apparatus and data collection

A double-beam UV–Vis spectrometer (UV-2401 PC, Shimadzu Co., Japan) was used for collecting absorptions in the UV–Vis range. Spectra were recorded quadruply and averaged for noise reduction. Samples were placed in quartz cells with 20 mm path length and recorded digitally in the wavelength range 345–570 nm in 0.1 nm steps with a slit width of 1 nm.

Determination of pH was made by a glass combination electrode (ELMETRON pH-meter Cp-315 Co., Zabrze, Poland) following the 5-point calibration scheme described by IUPAC [41, 42]. The calibration pH standard solution was traceable material (Merck Co., Darmstadt, Germany).

### Data analysis

Speciation was made with the geochemical code PREEEQC [43] and the probabilistic speciation code LJUNGSKILE [44]. Geochemical modelling is based on the parameters given in Table 1. Thermodynamic data, if not given otherwise, are taken from the JESS Thermodynamic Database [45, 46]. Spectral deconvolutions are made with custom-made codes based on the sequential Simplex [47] using least-squares criteria. Variance–covariance matrices and uncertainties of the spectral curves are estimated from quadratic forms in the minimum of the numerical optimum [48]. If not stated otherwise, uncertainties are given as 68 % confidence intervals. For data derived from small sample sizes, the necessary corrections have been made to transform standard deviations into confidence regions. The uncertainties in spectral decompositions (cf. Figs. 5, 6, 7) are given on the 0.95 ‰ range. None of the uncertainties represents a complete measurement uncertainty budget since only statistical contributions to uncertainty (e.g. misfit) are considered.
